# Obesity is common in chronic kidney disease and associates with greater antihypertensive usage and proteinuria: evidence from a cross‐sectional study in a tertiary nephrology centre

**DOI:** 10.1111/cob.12402

**Published:** 2020-08-26

**Authors:** William P. Martin, Jessica Bauer, John Coleman, Ludmilla Dellatorre‐Teixeira, Janice L.V. Reeve, Patrick J. Twomey, Neil G. Docherty, Aisling O'Riordan, Alan J. Watson, Carel W. le Roux, John Holian

**Affiliations:** ^1^ Diabetes Complications Research Centre Conway Institute of Biomolecular and Biomedical Research, School of Medicine, University College Dublin Dublin Ireland; ^2^ Department of Nephrology St. Vincent's University Hospital Dublin Ireland; ^3^ Department of Clinical Chemistry St. Vincent's University Hospital Dublin Ireland; ^4^ Institute of Clinical Sciences, Sahlgrenska Academy, University of Gothenburg Gothenburg Sweden; ^5^ Division of Investigative Science Imperial College London London UK

**Keywords:** chronic kidney disease, diabetes mellitus, diabetic kidney disease, obesity, overweight

## Abstract

Obesity is a treatable risk factor for chronic kidney disease progression. We audited the reporting of body‐mass index in nephrology outpatient clinics to establish the characteristics of individuals with obesity in nephrology practice. Body‐mass index, clinical information and biochemical measures were recorded for patients attending clinics between 3^rd^ August, 2018 and 18^th^ January, 2019. Inferential statistics and Pearson correlations were used to investigate relationships between body‐mass index, type 2 diabetes, hypertension and proteinuria. Mean ± SD BMI was 28.6 ± 5.8 kg/m^2^ (n = 374). Overweight and obesity class 1 were more common in males (*P* = .02). Amongst n = 123 individuals with obesity and chronic kidney disease, mean ± SD age, n (%) female and median[IQR] eGFR were 64.1 ± 14.2 years, 52 (42.3%) and 29.0[20.5] mL/min/BSA, respectively. A positive correlation between increasing body‐mass index and proteinuria was observed in such patients (*r* = 0.21, *P* = .03), which was stronger in males and those with CKD stages 4 and 5. Mean body‐mass index was 2.3 kg/m^2^ higher in those treated with 4‐5 versus 0‐1 antihypertensives (*P* = .03). Amongst n = 59 patients with obesity, chronic kidney disease and type 2 diabetes, 2 (3.5%) and 0 (0%) were prescribed a GLP‐1 receptor analogue and SGLT2‐inhibitor, respectively. Our data provides a strong rationale not only for measuring body‐mass index but also for acting on the information in nephrology practice, although prospective studies are required to guide treatment decisions in people with obesity and chronic kidney disease.

**What is already known about this subject**

- Obesity is recognized to exacerbate proteinuria amongst people with chronic kidney disease. The magnitude of this relationship, and how it is influenced by gender and chronic kidney disease stage, is inadequately described. In addition, whether or not a continuous relationship exists between increasing BMI and proteinuria amongst people with obesity and chronic kidney disease is unknown.
- Data on the incorporation of GLP‐1 receptor analogues and SGLT2‐inhibitors, which have metabolic, weight and renal benefits in people with type 2 diabetes, into real‐world clinical practice for the management of high‐risk patients with chronic kidney disease, are lacking.
- Outside of NHANES surveys in the United States, reports on the prevalence of obesity and associated comorbidity burden amongst people with chronic kidney disease are sparse. In addition, insights from such data are limited by the lack of granular information on physician‐assigned diagnoses of chronic kidney disease aetiology.

**What this study adds**

- Obesity is over‐represented in people with chronic kidney disease compared with background rates in the general population. Over 60% of the chronic kidney disease burden amongst people with obesity is attributable to diabetic kidney disease and hypertensive nephropathy; conversely, alternative chronic kidney disease aetiologies, including primary glomerular diseases, obstructive nephropathy and interstitial renal diseases, remain common in people with obesity and account for over 35% of chronic kidney disease cases in this setting.
- Amongst people with obesity and chronic kidney disease, increasing BMI associates with greater proteinuria and antihypertensive usage. The relationship between increasing BMI and proteinuria is stronger in males and those with advanced chronic kidney disease (stages 4 and 5). As a means of reducing the severity of hypertension and proteinuria, and consequently accelerated chronic kidney disease progression, intentional weight loss strategies should be explored in these particularly high‐risk subgroups of people with obesity and chronic kidney disease.
- The majority of people with obesity, chronic kidney disease and type 2 diabetes in our cohort were treated with weight‐promoting diabetes therapy (insulin and sulphonylureas), rather than newer therapies with weight‐lowering effects as well as established end‐organ benefits in the kidney (GLP‐1 receptor analogues and SGLT2‐inhibitors). In our study cohort reflective of real‐world contemporary nephrology practice, obesity was infrequently addressed as a modifiable risk factor for chronic kidney disease progression.

## 1 BACKGROUND

Obesity, hypertension and type 2 diabetes mellitus (T2DM) constitute inter‐related pandemics that have increased the prevalence of chronic kidney disease (CKD).^1^ Diabetic kidney disease (DKD) and obesity‐related glomerulopathy (ORG) are the two main drivers of CKD in people with obesity.^2^ DKD develops in approximately 40% of people with T2DM, with higher prevalence amongst non‐Caucasian ethnicities.^3^ DKD is associated with significant increases in cardiovascular and all‐cause mortality, with the majority of excess cardiovascular and all‐cause mortality attributable to diabetes occurring in those with kidney disease.^4^ ORG is a distinct cause of CKD characterized by sub‐nephrotic range proteinuria, glomerulomegaly and progressive renal functional loss.^2^ In the absence of routine histopathological confirmation of CKD aetiology, the true prevalence of ORG is unknown, although 4% to 10% of people with obesity have significant proteinuria (≥1+ by urine dipstick or uACR ≥30 mg/mmol).^2^

Reducing the severity of obesity with metabolic surgery decreases the incidence of albuminuria and end‐stage kidney disease (ESKD) over long‐term follow‐up.^5^, ^6^, ^7^ In a single‐centre study of 105 individuals with type 2 diabetes and albuminuria who underwent gastric bypass surgery, median reductions in albuminuria of 80.7% were achieved over mean 13‐month follow‐up.^8^ Postoperative reductions in proteinuria occur independently of improvements in blood pressure and metabolic control, suggesting that weight‐ and glycaemia‐independent mediators may contribute to the renoprotective effects of the procedure.^9^, ^10^ GLP‐1 receptor analogues (GLP1RAs) and sodium‐glucose cotransporter‐2 inhibitors (SGLT2is), which modify the course of DKD independently of their antihyperglycaemic properties, may improve CKD outcomes for people with obesity and/or T2DM.^11^, ^12^ In addition, integrated diabetology and nephrology care slows the progression of CKD in people with diabetes.^13^ Given the increasing recognition of obesity as an important driver of the onset and progression of CKD in people with and without T2DM,^1^, ^2^ and recent advances in medical and surgical treatment approaches to obesity,^14^, ^15^ there exists a rationale to investigate the impact of multi‐disciplinary clinics, which emphasize intentional weight loss on CKD progression and cardiovascular mortality in people with obesity. Prior to establishing such a clinic, we aimed to understand associations between obesity, CKD and other obesity complications in individuals with obesity attending general nephrology clinics at our tertiary referral centre.

## 2 MATERIALS AND METHODS

### 2.1 Study cohort

Permission was obtained from St Vincent's Healthcare Group, Dublin, Ireland for a prospective clinical audit (reference number 2018/2244) evaluating characteristics of people with obesity attending nephrology outpatient clinics. Under clinical audit guidelines, informed consent from individual participants was not obtained, but all data were handled as per the General Data Protection Regulation guidelines (EU), 2016/679. All procedures performed were in accordance with the ethical standards of the institutional audit committee and with the 1964 Helsinki declaration and its later amendments. Amongst people with obesity and CKD, cross‐sectional relationships between BMI, Chronic Kidney Disease‐Epidemiology Collaboration (CKD‐EPI) estimated glomerular filtration rate (eGFR), urine protein‐to‐creatinine ratio (uPCR), and usage of antihypertensives and glucose‐lowering medications are also reported. People with ESKD on haemodialysis or with a prior kidney transplant were excluded. Pregnant women, women in the post‐partum period for less than 3 months and individuals who self‐reported anabolic steroid use or with missing clinical and laboratory data were excluded.

### 2.2 Clinical information

Body height and weight were routinely measured in nephrology outpatient clinics between 3^rd^ August, 2018 and 18^th^ January, 2019 using a Seca 701 electronic scale and Seca 220 stadiometer, respectively. Body‐mass index (BMI) (kg/m^2^) was calculated: body weight (kg)/(height × height [m^2^]).^16^ Only the first BMI measurement was considered for individuals that attended nephrology clinics on multiple occasions during the data collection period. BMI was categorized according to the World Health Organization criteria.^16^ Clinical information (demographics, diabetes type, diabetes complications, office blood pressure, cardiovascular comorbidities and medication usage) was recorded from hospital outpatient and discharge records at or before the study entry date. CKD was defined according to 2012 KDIGO consensus criteria, including impaired glomerular filtration (eGFR <60 mL/min/BSA) or proteinuria sustained for ≥3 months, or abnormal renal histopathology or abnormal renal imaging.^17^ CKD aetiology was manually abstracted from nephrology outpatient records. Patients with diabetes and CKD were presumed to have DKD unless clear evidence of an additional or alternative CKD aetiology was present. Patients with obesity, hypertension and CKD in the absence of diabetes were classified as hypertensive nephropathy. Primary glomerular diseases were recorded as the principal CKD aetiology only in the presence of renal histopathological confirmation. Diagnoses of autosomal dominant polycystic kidney disease (ADPKD) and obstructive nephropathy were assigned only with radiological confirmation. Chronic pyelonephritis and NSAID‐ and lithium‐related CKD were collectively categorized as interstitial renal disease. In the absence of supportive renal histopathological evidence, ORG was not assigned as a distinct CKD aetiology.

### 2.3 Biochemical measures

All recorded clinical biochemistry tests (urine/serum creatinine, urine total protein, serum lipids, haematinics and glycated haemoglobin [HbA_1c_]) were analysed via routine clinical biochemistry services at St Vincent's University Hospital, Dublin. Haemoglobin was measured and reported by the Haematology laboratory. The Pathology Department is accredited by the Irish National Accreditation Board to ISO 15189:2012 standards. All analyses, with the exception of haemoglobin and HbA_1c_, were performed on the Roche/Hitachi Cobas 8000 analyser series; c702 and e602 (Roche Diagnostics GmbH, Mannheim, Germany). The enzymatic creatinine method was used, traceable to serum reference manual 914 and isotope‐dilution mass spectrometry. HbA_1c_ was measured by high‐performance liquid chromatography (A. Menarini Diagnostics HA‐8180 V, Florence, Italy). Haemoglobin was measured on the Sysmex XE‐5000 differential analyser (Sysmex Europe GmbH, Norderstedt, Germany). Urine protein was measured using turbidimetry with benzethonium chloride, traceable to a NIST standard. CKD‐EPI eGFR, calculated using standard formulae, was recorded and expressed as mL/min/body surface area (BSA).^18^ uPCR was calculated and expressed as mg/mmol. LDL‐cholesterol (mmol/L) was calculated using the Friedewald equation. Transferrin saturation (%) was calculated as: ((iron)/(transferrin) × 22.75) × 100. Laboratory data were manually extracted from the hospital laboratory information system (LIS; DXC APEX) on the study entry date; if unavailable, laboratory results within 12 months before or after the study entry date were extracted from LIS using COGNOS Impromptu version 7.1.529.0 (Cognos Incorporated 2003).

### 2.4 Statistical analyses

RStudio version 3.6.1 was used for analysis. BMI distribution and prevalence of BMI categories at study enrolment were summarized by descriptive statistics. Categorical variables are presented as frequencies and percentages and were compared between groups using *χ* ^2^ tests. Fisher's exact tests were used to compare categorical variables between groups when *χ* ^2^ test assumptions were violated (frequency of values <5 in ≥1 cell of contingency table). Continuous variables with normal and skewed distributions are presented as mean ± SD and median [interquartile range], respectively. One‐way between‐group ANOVAs and Kruskal‐Wallis tests were used to assess for differences across the BMI categories of obesity (three groups) in continuous variables with normal and skewed distributions, respectively. Independent sample *t* tests and Wilcoxon rank‐sum tests were used to assess for gender differences (two groups) in continuous variables with normal and skewed distributions, respectively. Univariate relationships between BMI, uPCR and HbA_1c_ were investigated by Pearson correlations. The functions “ggscatter” and “ggboxplot” from the R package “ggpubr” were used to generate scatterplots and boxplots, respectively.^19^ *P* <.05 was considered statistically significant.

## 3 RESULTS

### 3.1 Overweight and obesity are common amongst people attending nephrology clinics

**FIGURE 1:**
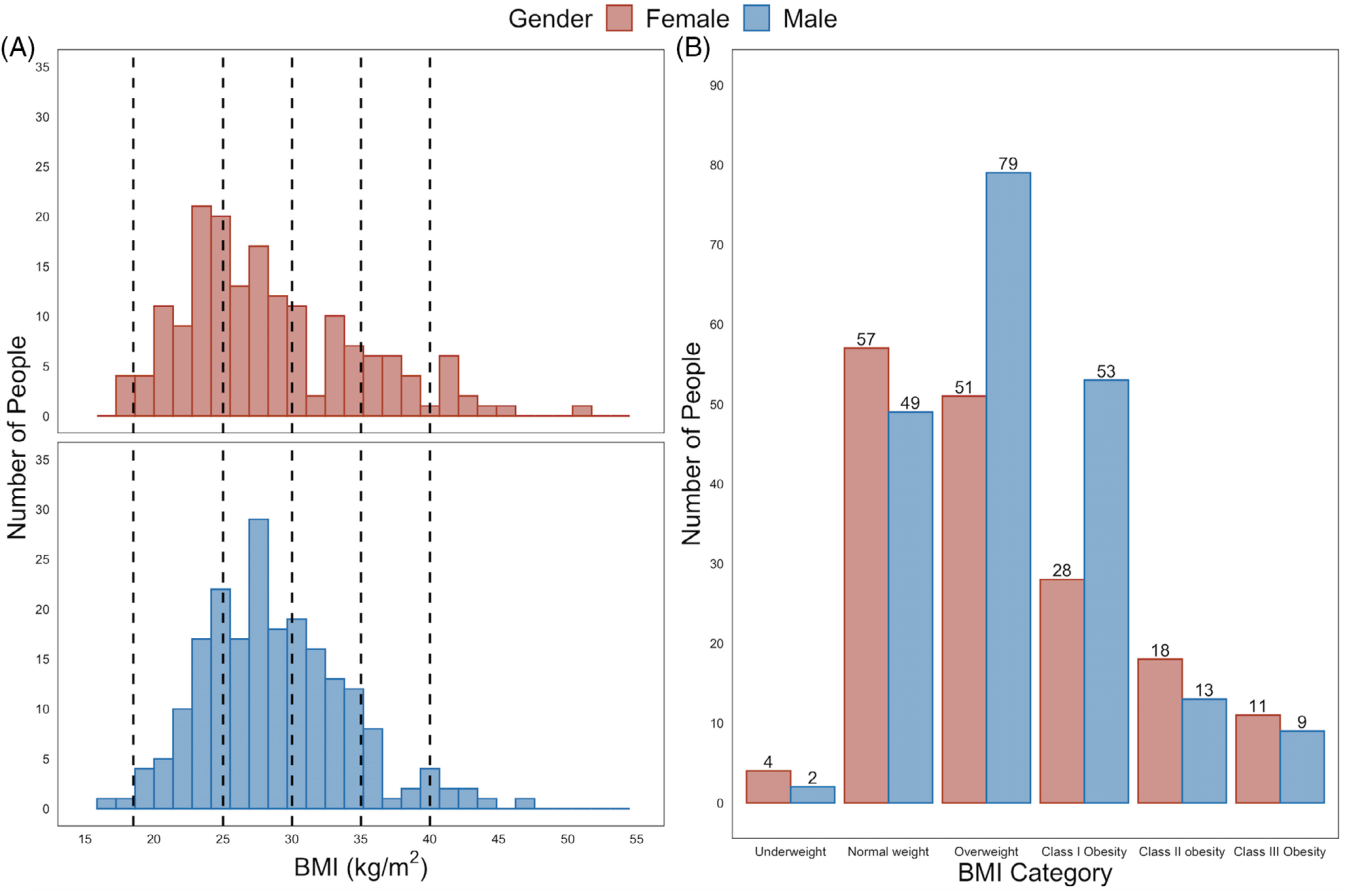
BMI distribution of people attending outpatient nephrology clinics, stratified by gender. Panel A: Histograms of BMI distribution stratified by gender. Black vertical dashed lines on the x‐axis (BMI) demarcate cutoffs for defining WHO BMI categories at 18.5, 25, 30, 35 and 40 kg/m^2^. Panel B: Frequency of WHO BMI categories by gender. Definition of WHO BMI categories is as follows: underweight (<18.5 kg/m^2^), normal weight (18.5‐24.9 kg/m^2^), overweight (25‐29.9 kg/m^2^), obesity class 1 (30‐34.9 kg/m^2^), obesity class 2 (35‐39.9 kg/m^2^), and obesity class 3 (≥40 kg/m^2^). Females are shaded in red, males in blue

BMI was measured in 384 individuals over the 6‐month study period; n = 10 people were excluded for: active haemodialysis treatment (n = 3), missing clinical and laboratory data (n = 3), pregnancy (n = 2), <3 months post‐partum (n = 1), and anabolic steroid use (n = 1). Table 1 summarizes the demographic and anthropometric characteristics of the remaining 374 individuals included in downstream analysis. Mean age of the study cohort was 59.0 ± 18.1 years, with 169 females (45.2%) and 358 Caucasians (95.7%). Compared to males, females were 3.5 years younger (*P* = .06) and had a 1.3% higher prevalence of Caucasian ethnicity (*P* = .07). Mean BMI was 28.6 ± 5.8 kg/m^2^, and did not significantly differ between males and females (*P* = .61). Prevalent BMI distributions were: <18.5 kg/m^2^ (n = 6, 1.6%), 18.5 to 24.9 kg/m^2^ (n = 106, 28.3%), 25 to 29.9 kg/m^2^ (n = 130, 34.8%), 30 to 34.9 kg/m^2^ (n = 81, 21.7%), 35 to 39.9 kg/m^2^ (n = 31, 8.3%), ≥40 kg/m^2^ (n = 20, 5.3%). Overall, n = 132 individuals (35.3%) were affected by obesity, compared to a population average in Ireland of 23%.^20^ A higher proportion of men than women had class 1 obesity; conversely, women were more likely than men to be of normal weight or have obesity classes 2 and 3 (*P* = .02). Figure 1 presents histograms and bar charts of BMI distribution stratified by gender, illustrating the differences in BMI distribution between males and females, which were present despite a similar mean BMI in both genders.

**TABLE 1:** Baseline characteristics of the study cohort (n = 374)

Characteristic	Data available (n (%))	Total cohort (n = 374)	Males (n = 205)	Females (n = 169)	*P*
Age (mean ± SD; years)^a^	374 (100)	59.0 ± 18.1	60.6 ± 17.8	57.1 ± 18.2	.06
Gender (n (%))	374 (100)				N/A
Male		205 (54.8)	205 (100)	0 (0)	
Female		169 (45.2)	0 (0)	169 (100)	
Ethnicity (n (%))^b^	374 (100)				.06
Caucasian		358 (95.7)	195 (95.1)	163 (96.4)	
Asian		8 (2.1)	7 (3.4)	1 (0.6)	
Latin American		5 (1.3)	3 (1.5)	2 (1.2)	
African American		3 (0.8)	0 (0)	3 (1.8)	
Anthropometry	374 (100)				
Body weight (mean ± SD; kg)		80.6 ± 17.8	86.0 ± 16.4	74.1 ± 17.2	**<.001**
Body height (mean ± SD; m)		1.68 ± 0.10	1.73 ± 0.08	1.62 ± 0.08	**<.001**
Body‐mass index (mean ± SD; kg/m^2^)		28.6 ± 5.8	28.8 ± 5.2	28.4 ± 6.4	0.61
BMI classes	374 (100)				**.02**
Underweight (<18.5 kg/m^2^)		6 (1.6)	2 (1.0)	4 (2.4)	
Normal weight (18.5‐24.9 kg/m^2^)		106 (28.3)	49 (23.9)	57 (33.7)	
Overweight (25.0‐29.9 kg/m^2^)		130 (34.8)	79 (38.5)	51 (30.2)	
Class 1 obesity (30.0‐34.9 kg/m^2^)		81 (21.7)	53 (25.9)	28 (16.6)	
Class 2 obesity (35.0‐39.9 kg/m^2^)		31 (8.3)	13 (6.3)	18 (10.7)	
Class 3 obesity (≥40 kg/m^2^)		20 (5.3)	9 (4.4)	11 (6.5)	

*Note*: Values are given as n (%) for categorical variables, or mean ± SD for normally distributed continuous variables unless otherwise indicated. *P* <.05 was considered statistically significant.

Abbreviations: N/A, not applicable; SD, standard deviation.

^a^ Independent samples *t*‐test was used to assess for variation in normally distributed continuous variables by gender.

^b^ *χ* ^2^ analysis or Fisher's exact test was used to analyse for differences in categorical variables by gender.

### 3.2 Clinical and laboratory characteristics of individuals with obesity attending nephrology clinics

In total, 132 people with obesity (BMI ≥ 30 kg/m^2^) attended nephrology clinics during the study period. Nine patients did not have confirmed CKD. Of these, four were attending nephrology clinics for blood pressure management, two for evaluation of high serum creatinine, one for evaluation of proteinuria, one for nephrolithiasis and one for recurrent urinary tract infections. Table 2 summarizes the clinical characteristics of the remaining n = 123 individuals with obesity and CKD, stratified by obesity class. As observed in the cohort as a whole, males were more likely to have obesity class 1 while females were more likely to have obesity classes 2 and 3 (*P* = .04). The majority of the cohort had hypertension (87%) and dyslipidaemia (63%), while almost 25% had established coronary artery disease. Over 60% and 50% of people were treated with renin‐angiotensin‐aldosterone system (RAAS) blockade and statins, respectively. Overall, 50 (40.7%) patients had DKD and together, DKD and hypertensive nephropathy accounted for almost 65% of cases of CKD. Conversely, alternative CKD aetiologies remained common in people with obesity and accounted for over 35% of CKD cases. Often, individuals with obesity had advanced CKD; stage 4 was the most common CKD class and median eGFR was 29 [20.5] mL/min/BSA. No significant differences in office blood pressure, metabolic parameters (HbA_1c_ and lipid indices), anaemia parameters (haemoglobin and iron indices), cardiovascular comorbidities, CKD stage and aetiology, degree of proteinuria and medication usage were observed across categorical obesity classes.

**TABLE 2:** Characteristics of individuals with obesity (BMI ≥ 30 kg/m^2^) and chronic kidney disease attending nephrology clinics, stratified by obesity class (*n* = 123)

Characteristic	Data available (n (%))	All obesity classes (BMI ≥ 30 kg/m^2^) (n = 123)	Class 1 obesity (BMI 30‐34.9 kg/m^2^) (n = 75)	Class 2 obesity (BMI 35‐39.9 kg/m^2^) (n = 30)	Class 3 obesity (BMI ≥ 40 kg/m^2^) (n = 18)	*P*
Age (mean ± SD; years)^a^	123 (100)	64.1 ± 14.2	65.1 ± 14.6	63.5 ± 13.8	60.6 ± 13.5	.47
Female (n (%))^b^	123 (100)	52 (42.3)	25 (33.3)	17 (56.7)	10 (55.6)	**.04**
Caucasian (n (%))	123 (100)	121 (98.4)	74 (98.7)	30 (100)	17 (94.4)	.33
Body‐mass index (mean ± SD; kg/m^2^)	123 (100)	34.9 ± 4.1	32.3 ± 1.5	36.7 ± 1.4	42.6 ± 2.8	**<.001**
Blood pressure (mean ± SD; mmHg)	123 (100)					
Systolic		140.3 ± 21.6	138.0 ± 19.5	142.0 ± 24.6	147.0 ± 23.9	.29
Diastolic		79.4 ± 12.5	78.9 ± 11.1	79.7 ± 12.6	80.9 ± 17.6	.82
Comorbidities (n (%))						
Hypertension	121 (98.4)	105 (86.8)	64 (85.3)	25 (86.2)	16 (94.1)	.69
Dyslipidaemia	119 (96.7)	75 (63.0)	51 (68.9)	17 (58.6)	7 (43.8)	.14
Coronary artery disease	122 (99.2)	28 (23.0)	14 (18.7)	11 (37.9)	3 (16.7)	.10
Cerebrovascular disease	123 (100)	12 (9.8)	8 (10.7)	2 (6.7)	2 (11.1)	.83
Peripheral arterial disease	123 (100)	12 (9.8)	9 (12.0)	3 (10.0)	0 (0)	.38
Diabetes mellitus	123 (100)	62 (50.4)	37 (49.3)	14 (46.7)	11 (61.1)	.60
Type 1 (n (%))		3 (2.4)	2 (2.7)	1 (3.3)	0 (0)	1
Type 2 (n (%))		59 (48.0)	35 (46.7)	13 (43.3)	11 (61.1)	.46
Metabolic parameters						
Glycated haemoglobin (mean ± SD; mmol/mol)	107 (87.0)	50.3 ± 19.0	52.7 ± 22.0	44.7 ± 11.7	49.6 ± 13.8	.19
Total cholesterol (mean ± SD; mmol/L)	117 (95.1)	4.6 ± 1.4	4.6 ± 1.4	4.5 ± 1.4	4.9 ± 1.5	.59
Low‐density lipoprotein cholesterol (mean ± SD; mmol/L)	111 (90.2)	2.4 ± 1.2	2.3 ± 1.1	2.5 ± 1.3	2.8 ± 1.2	.29
High‐density lipoprotein cholesterol (mean ± SD; mmol/L)	117 (95.1)	1.3 ± 0.5	1.4 ± 0.5	1.2 ± 0.4	1.2 ± 0.4	.33
Triglycerides (median [IQR]; mmol/L)^c^	117 (95.1)	1.7 [1.1]	1.8 [1.1]	1.7 [1.0]	2.0 [1.4]	.57
Anaemia parameters						
Haemoglobin (mean ± SD; g/dL)	123 (100)	12.2 ± 1.8	12.3 ± 1.8	12.0 ± 1.8	12.0 ± 1.8	.76
Ferritin (median [IQR]; μg/L)	114 (92.7)	154.0 [210.8]	156.0 [188.0]	164.0 [264.0]	97.0 [282.0]	.62
Transferrin saturation (mean ± SD; %)	119 (96.7)	23.6 ± 9.8	24.6 ± 9.4	22.5 ± 9.3	20.8 ± 12.1	.29
Chronic kidney disease (n (%))	123 (100)	123 (100)	75 (100)	30 (100)	18 (100)	N/A
eGFR (median [IQR]; mL/min/BSA)	123 (100)	29.0 [20.5]	31.0 [19.5]	27.0 [20.2]	28.5 [22.8]	1
uPCR (median [IQR]; mg/mmol)	109 (88.6)	60.0 [211.0]	60.0 [188.0]	65.5 [220.0]	98.0 [242.0]	.35
Chronic kidney disease stage (n (%))						.13
Grade 1 (eGFR ≥90)		3 (2.4)	3 (4.0)	0 (0)	0 (0)	
Grade 2 (eGFR 60‐89)		6 (4.9)	1 (1.3)	4 (13.3)	1 (5.6)	
Grade 3a (eGFR 45‐59)		20 (16.3)	13 (17.3)	3 (10.0)	4 (22.2)	
Grade 3b (eGFR 30‐44)		32 (26.0)	23 (30.7)	5 (16.7)	4 (22.2)	
Grade 4 (eGFR 15‐29)		50 (40.7)	26 (34.7)	17 (56.7)	7 (38.9)	
Grade 5 (eGFR <15)		12 (9.8)	9 (12.0)	1 (3.3)	2 (11.1)	
Principal CKD aetiology (n (%))	123 (100)					.86
Diabetic kidney disease		50 (40.7)	29 (38.7)	12 (40.0)	9 (50.0)	
Hypertensive nephropathy		29 (23.6)	20 (26.7)	6 (20.0)	3 (16.7)	
Primary glomerular disease		18 (14.6)	8 (10.7)	6 (20.0)	4 (22.2)	
Interstitial renal disease		8 (6.5)	4 (5.3)	2 (6.7)	2 (11.1)	
ADPKD		5 (4.1)	3 (4.0)	2 (6.7)	0 (0)	
Obstructive nephropathy		5 (4.1)	4 (5.3)	1 (3.3)	0 (0)	
Post‐acute kidney injury		5 (4.1)	5 (6.7)	0 (0)	0 (0)	
Calcineurin inhibitor nephrotoxicity		2 (1.6)	1 (1.3)	1 (3.3)	0 (0)	
Atrophic kidney		1 (0.8)	1 (1.3)	0 (0)	0 (0)	
Non‐diabetes medications						
Either ACE‐inhibitor or ARB (n (%))	123 (100)	76 (61.8)	44 (58.7)	21 (70.0)	11 (61.1)	.56
Number of antihypertensives (median [IQR])	123 (100)	3.0 [2.0]	2.0 [2.0]	2.0 [3.0]	3.0 [1.5]	.20
Statin (n (%))	122 (99.2)	66 (54.1)	43 (58.1)	17 (56.7)	6 (33.3)	.16

*Note*: Values are given as n (%) for categorical variables, or mean ± SD for normally distributed continuous variables, unless otherwise indicated. Median [IQR] values are presented for continuous variables that are not normally distributed. *P* <.05 was considered statistically significant.

Abbreviations: ACE, angiotensin‐converting enzyme; ADPKD, autosomal dominant polycystic kidney disease; ARB, angiotensin‐II receptor blocker; CKD, chronic kidney disease; eGFR, estimated glomerular filtration rate; IQR, interquartile range; N/A, not applicable; SD, standard deviation; uPCR, urine protein‐to‐creatinine ratio.

^a^ One‐way between‐groups ANOVA was used to assess for variation in normally distributed continuous variables across obesity classes.

^b^ *χ* ^2^ analysis or Fisher's exact test was used to analyse for differences in categorical variables across obesity classes.

^c^ Kruskal‐Wallis test was used to assess for variation across obesity classes in continuous variables that were not normally distributed.

### 3.3 Increasing BMI associates with higher uPCR and greater antihypertensive medication usage in individuals with obesity and CKD

**FIGURE 2:**
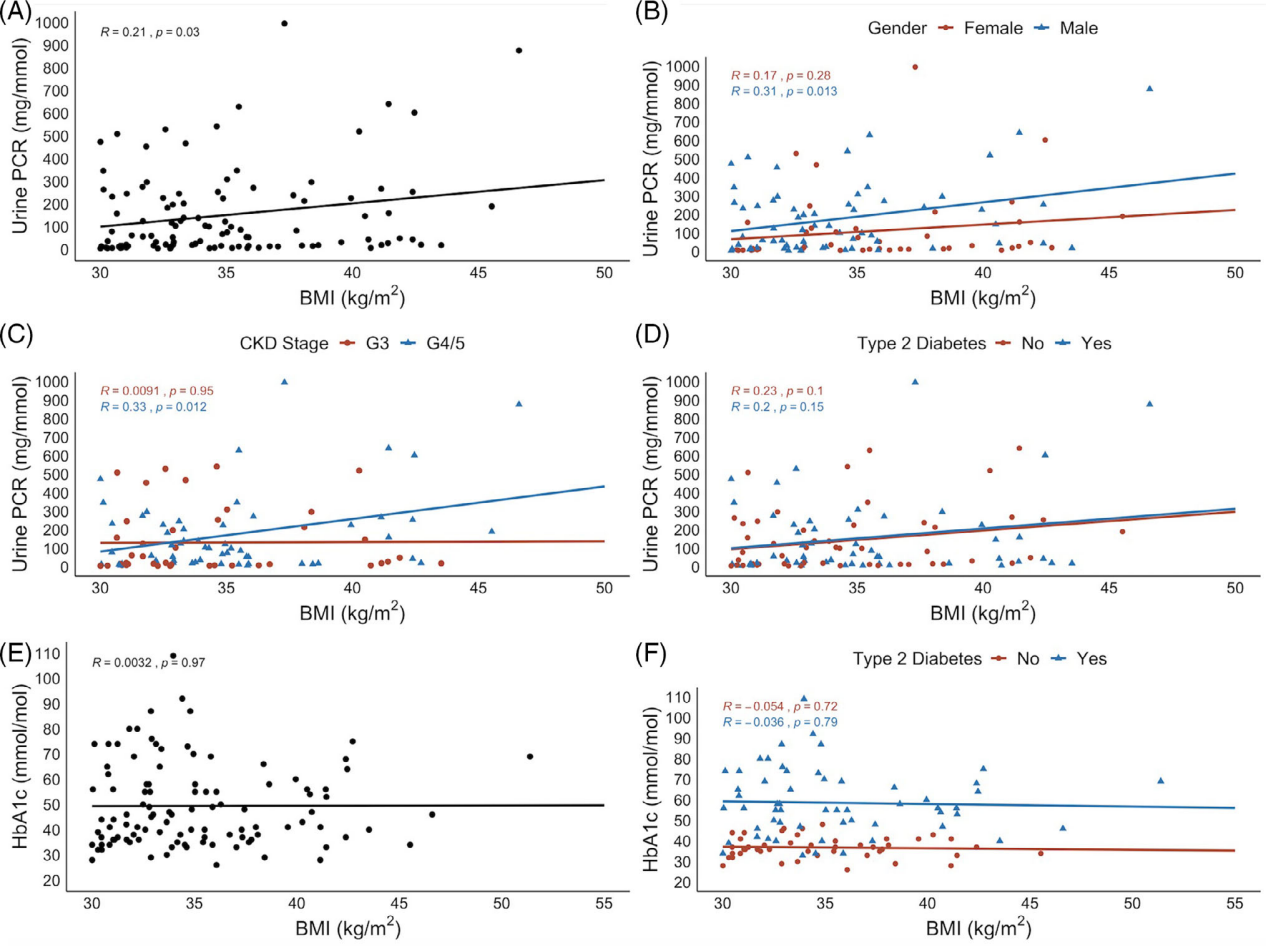
Cross‐sectional relationships between BMI, urine protein‐to‐creatinine ratio (uPCR) and glycated haemoglobin amongst people with obesity and CKD. Panel A: Scatterplot of BMI and uPCR reveals a modest positive correlation (*r* = 0.21, *P* = .03). Panel B: Scatterplot of BMI and uPCR stratified by gender. Panel C: Scatterplot of BMI and uPCR stratified by CKD stage. G3 = grade 3 (eGFR 30‐60 mL/min/BSA); G4/5 = grades 4 and 5 (eGFR <30 mL/min/BSA). Panel D: Scatterplot of BMI and uPCR stratified by type 2 diabetes mellitus status. Individuals with type 1 diabetes mellitus were removed from the non‐type 2 diabetes mellitus group in this plot. Panel E: Scatterplot of BMI and HbA_1c_ reveals no relationship between the two variables (*r* = 0.003, *P* = .97). Panel F: Scatterplot of BMI and HbA_1c_ stratified by type 2 diabetes mellitus status. Individuals with type 1 diabetes mellitus were removed from the non‐type 2 diabetes mellitus group in this plot. Reported *r* and *P* values are derived from Pearson correlations. *P* <.05 was considered statistically significant

**FIGURE 3:**
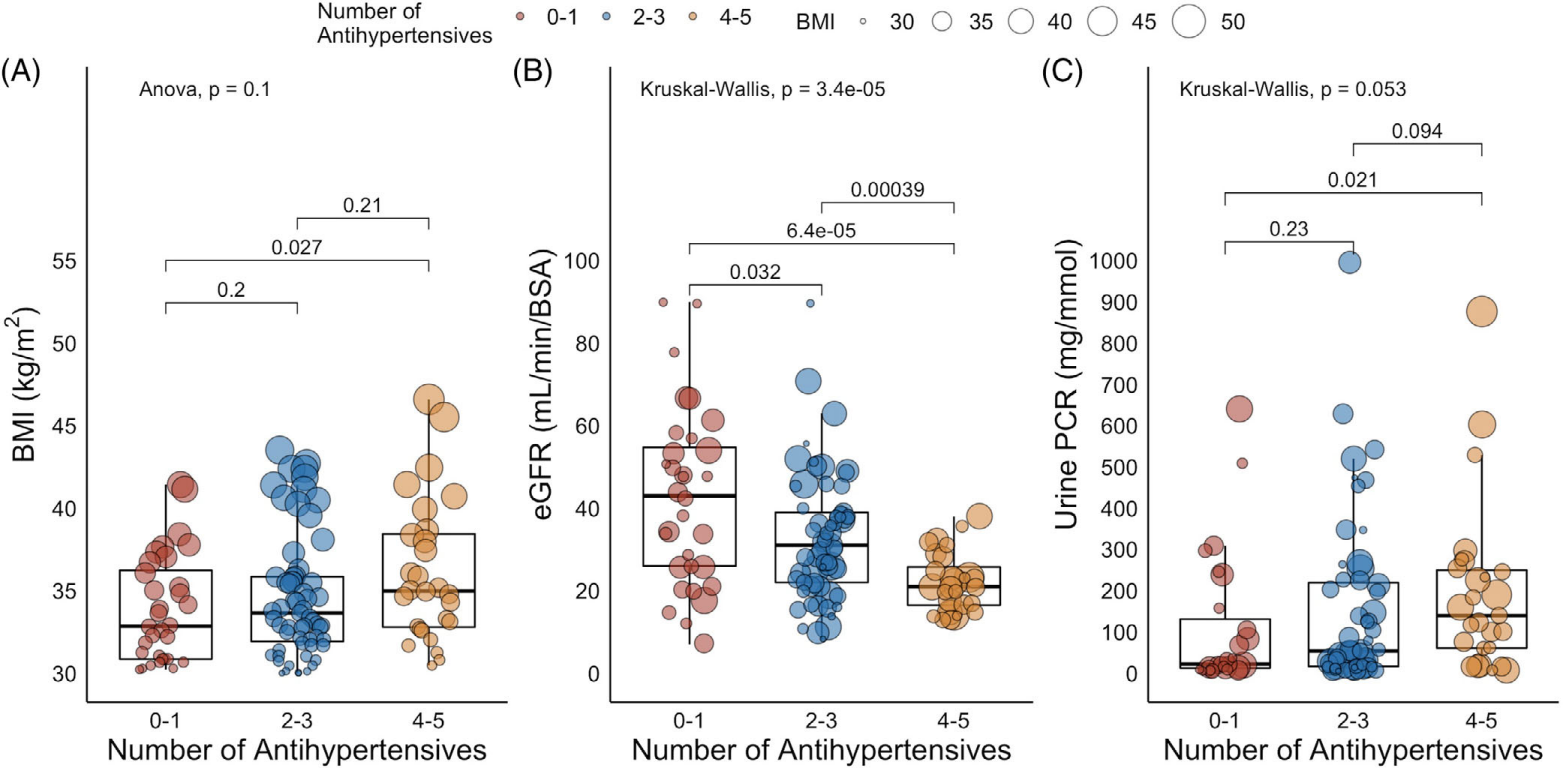
Cross‐sectional relationships between BMI and antihypertensive usage amongst people with obesity and CKD. Panel A: Boxplots of BMI stratified by the number of antihypertensive medications. Comparisons between three groups are made by ANOVA; comparisons between two groups by independent samples *t* test. Panel B: Boxplots of eGFR stratified by the number of antihypertensive medications. Comparisons between three groups are made by the Kruskal‐Wallis test; comparisons between two groups by Wilcoxon rank‐sum test. Panel C: Boxplots of uPCR stratified by the number of antihypertensive medications. Comparisons between three groups are made by the Kruskal‐Wallis test; comparisons between two groups by Wilcoxon rank‐sum test. The number of antihypertensive medications was categorized into three groups as follows: 0 to 1, 2 to 3 and 4 to 5. The size of individual data points is scaled by BMI, emphasizing the cross‐sectional associations between increasing BMI with increasing uPCR and antihypertensive usage. *P* <.05 was considered statistically significant

Figure 2 depicts the relationship between increasing BMI, uPCR and HbA_1c_ amongst people with obesity and CKD. When BMI was treated as a continuous variable, a modest positive correlation (*r* = 0.21, *P* = .03) was observed between increasing BMI and increasing uPCR in individuals with obesity. The relationship between BMI and uPCR was stronger in males (*r* = 0.31, *P* = .01) than females (*r* = 0.17, *P* = .28), although the smaller sample of females may have limited statistical power. Increasing BMI also had a greater impact on uPCR in those with CKD stages 4 and 5 (*r* = 0.33, *P* = .012). No relationship between BMI and HbA_1c_ was observed amongst all patients with obesity and CKD (*r* = 0.003, *P* = .97) and amongst the subgroup of patients with obesity, CKD and type 2 diabetes (*r* = −0.04, *P* = .79). Figure 3 illustrates the relationships between increasing BMI and antihypertensive usage in people with obesity and CKD. Figure 3, panel A highlights that compared with individuals on 0‐1 antihypertensives (BMI 33.8 ± 3.2 kg/m^2^), BMI was significantly higher in those treated with 4‐5 antihypertensives (BMI 36.1 ± 4.3 kg/m^2^, *P* = .03). Figure 3, panels B and C highlight that clear relationships between declining eGFR, increasing uPCR and increased antihypertensive medication usage were observed in people with obesity and CKD. Compared with individuals on 0‐1 antihypertensives, median eGFR was 22 mL/min/BSA lower (*P* < .001) and median uPCR was 118 mg/mmol higher (*P* = .02) in those treated with 4‐5 antihypertensives. The size of individual data points in Figure 3 is scaled by BMI to emphasize the point that increased BMI in people with CKD associates with increased uPCR and antihypertensive usage.

### 3.4 Individuals with obesity, T2DM and CKD are infrequently treated with weight‐neutral and weight‐lowering diabetes therapy

Table 3 presents details on medical therapy of diabetes, blood pressure, and proteinuria in people with obesity, CKD and T2DM, stratified by obesity class. No differences in medical therapy were observed across BMI classes. Almost 70% of such patients were treated with RAAS blockade, while over 60% were on a statin. Insulin and sulphonylureas were the two most commonly used glucose‐lowering medications in this setting (approximately 40% of patients were prescribed each of these drug classes). Over 25% of people were treated with a DPP4‐inhibitor, mostly linagliptin. Only 21% were prescribed metformin, consistent with concerns regarding lactic acidosis in advanced CKD.^21^ Only two people were treated with a GLP1RA and no patients were prescribed an SGLT2i. No patients with a BMI ≥40 kg/m^2^ were treated with either a GLP1RA or SGLT2i.

**TABLE 3:** Medical therapy of individuals with obesity (BMI ≥ 30 kg/m^2^), type 2 diabetes mellitus and chronic kidney disease attending nephrology clinics, stratified by obesity class (n = 59)

Characteristic	Data available (n (%))	All obesity classes (BMI ≥ 30 kg/m^2^) (n = 59)	Class 1 obesity (BMI 30–34.9 kg/m^2^) (n = 35)	Class 2 obesity (BMI 35–39.9 kg/m^2^) (n = 13)	Class 3 obesity (BMI ≥ 40 kg/m^2^) (n = 11)	*P*
Glycated haemoglobin (mean ± SD; mmol/mol)^a^	58 (98.3)	60.1 ± 19.7	63.3 ± 23.5	53.2 ± 10.0	57.1 ± 10.8	.27
Non‐diabetes medications	59 (100)					
Either ACE‐inhibitor or ARB (n (%))^b^		41 (69.5)	22 (62.9)	11 (84.6)	8 (72.7)	.36
Number of antihypertensives (mean ± SD)		2.9 ± 1.3	2.7 ± 1.3	3.2 ± 1.3	3.4 ± 1.0	.31
Statin (n (%))		37 (62.7)	23 (65.7)	9 (69.2)	5 (45.5)	.46
Diabetes medications						
Metformin (n (%))	58 (98.3)	12 (20.7)	6 (17.1)	3 (25.0)	3 (27.3)	.73
Sulphonylurea (n (%))	58 (98.3)	22 (37.9)	14 (40.0)	4 (33.3)	4 (36.4)	.93
DPP4i	58 (98.3)	16 (27.6)	10 (28.6)	3 (25.0)	3 (27.3)	1
GLP1RA (n (%))	58 (98.3)	2 (3.5)	1 (2.9)	1 (8.3)	0 (0)	.64
SGLT2i (n (%))	58 (98.3)	0 (0)	0 (0)	0 (0)	0 (0)	N/A
Insulin (n (%))	58 (98.3)	24 (41.4)	15 (42.9)	5 (41.7)	4 (36.4)	1
Number of glucose‐lowering medications (median [IQR])^c^	57 (96.6)	1.0 [1.0]	1.0 [1.0]	1.0 [0.5]	1.0 [1.0]	1

*Note*: Values are given as n (%) for categorical variables, or mean ± SD for normally distributed continuous variables unless otherwise indicated. Median [IQR] values are presented for continuous variables that are not normally distributed. *P* <.05 was considered statistically significant.

Abbreviations: ACE, angiotensin‐converting enzyme; ARB, angiotensin‐II receptor blocker; DPP4i, dipeptidyl peptidase‐4 inhibitor; GLP1RA, glucagon‐like peptide‐1 receptor analogue; IQR, interquartile range; N/A, not applicable; SD, standard deviation; SGLT2i, sodium‐glucose co‐transporter‐2 inhibitor.

^a^ One‐way between‐groups ANOVA was used to assess for variation in normally distributed continuous variables across obesity classes.

^b^ *χ^2^* analysis or Fisher's exact test was used to analyse for differences in categorical variables across obesity classes.

^c^ Kruskal‐Wallis test was used to assess for variation across obesity classes in continuous variables that were not normally distributed.

## 4 DISCUSSION

This study provides insight into the BMI distribution of males and females attending general nephrology clinics. Obesity (35.3%) was common in outpatient nephrology practice. Obesity class 1 was more common in men, while normal weight and obesity classes 2 and 3 were more frequently observed in females. Amongst individuals with obesity and CKD, a positive correlation was observed between increasing BMI and uPCR, a relationship, which was stronger in males and those with more advanced CKD stages (eGFR <30 mL/min/BSA). BMI was higher in individuals treated with 4‐5 compared with 0‐1 antihypertensives. Individuals with obesity, CKD and T2DM were frequently prescribed RAAS blockade, statins, insulin and sulphonylureas, but infrequently prescribed diabetes therapy with weight‐lowering effects, including GLP1RAs and SGLT2is.

Obesity was more common in people with CKD than expected based on trends in the general Irish population. In the 2015 Healthy Ireland survey of 6,142 people aged ≥15 years, the prevalence of obesity was 23%.^20^ An additional 12% of people attending nephrology clinics in Ireland are affected by obesity compared with the general population. The 2015 Healthy Ireland survey also identified that compared with men, women are more likely to be of normal weight (men: 31%, women: 44%) and less likely to be affected by overweight (men: 43%, women: 31%) or obesity (men: 25%, women 22%).^20^ Findings on gender differences in BMI distribution in our study thus mirror background patterns in the Irish population.^20^ Globally, obesity is more common in women than men and the majority of patients recruited to studies of metabolic surgery as a treatment for obesity have been female (approximately 70%).^22^, ^23^, ^24^

The prevalence of obesity amongst people with CKD is even higher in the United States, where it increased from 38.1% between 1999 and 2002 to 44.1% between 2011 and 2014.^25^ The prevalence of obesity is higher in the United States' general population, where it was 42.4% amongst adults during 2017 to 2018.^26^ The higher rate of obesity amongst patients with CKD in the United States thus at least partly reflects a higher background prevalence of obesity in the general population than in Ireland. Overweight and obesity are the norm rather than the exception in nephrology practice both in Ireland and internationally. Given obesity's tight association with the two most common causes of CKD, T2DM and hypertension,^1^ as well as its independent relationship with ORG and subsequent progression to ESKD,^2^ there exists a strong rationale to investigate the role of intentional weight loss strategies on CKD progression.

Amongst people with obesity and CKD, the complications of hypertension, dyslipidaemia and diabetes mellitus were very common and present in over 85%, 60% and 50% of patients, respectively. No major differences in cardiovascular comorbidities or CKD stage were observed across incremental categorical classes of obesity. This may be partly related to the relatively low number of patients (n = 18) with obesity class 3 (BMI ≥ 40 kg/m^2^) and CKD. Obesity‐related glomerular hyperfiltration, which is an independent predictor of adverse cardiovascular outcomes, and the obesity paradox may have contributed to the high burden of cardiovascular disease even amongst individuals with less severe obesity (WHO classes I and II) in our cohort.^27^, ^28^ However, a linear relationship between BMI and risk of ORG does not exist in people with obesity. Almost half the total number of biopsy‐proven cases of ORG occurred in those with BMI <40 kg/m^2^ in a single‐centre series from the United States.^29^ Thus, as the complication burden amongst people with obesity and CKD is similar across obesity classes, and obesity classes 1 and 2 are much more prevalent than obesity class 3, it is plausible that the maximum benefit of intentional weight loss strategies in nephrology practice may be achieved in those with obesity classes 1 and 2. Indeed, the Microvascular Outcomes after Metabolic Surgery study, which randomized individuals with T2DM and microalbuminuria to medical therapy or medical therapy plus metabolic surgery, selectively recruited individuals with class 1 obesity.^30^ Further prospective studies of intentional weight loss strategies across the spectrum of obesity severity in people with CKD are required.

Despite the lack of observed difference in CKD severity across obesity classes 1 to 3 in our cohort, when BMI was treated as a continuous variable, a significant positive correlation between increasing BMI and increasing uPCR was observed amongst people with obesity and CKD. Importantly, this association was stronger in males and those with CKD stages 4 and 5. Mean BMI was also 2.3 kg/m^2^ higher in those treated with 4 to 5 compared with 0 to 1 antihypertensives. These findings suggest the presence of a continuous gradient between increasing BMI and increasing severity of proteinuria and hypertension, the two major modifiable determinants of CKD progression,^31^ in people with obesity, which was likely diminished by categorizing BMI according to WHO obesity classes. While increased BMI confers a survival advantage in people on haemodialysis,^32^ the association between increasing BMI and proteinuria in our study, particularly in those with CKD stages 4 and 5, suggests that obesity may accelerate progression to ESKD in those with advanced CKD. Those with eGFR <30 mL/min/BSA are typically excluded from many interventional studies of new therapies.^33^, ^34^ Given the significant annual healthcare costs associated with the treatment of ESKD,^35^ as well as associated decrements in quality of life,^36^ studies of intentional weight loss to minimize obesity‐associated proteinuria and consequent accelerated CKD progression and cardiovascular mortality in those with CKD stages 4 and 5 should be a priority from both health economic and quality of life perspectives. In addition, recipient obesity increases the risk of kidney allograft complications and is a major barrier to kidney transplantation; most transplant centres consider a BMI ≥35 kg/m^2^ a relative contraindication to kidney transplantation.^37^, ^38^ Studies of intentional weight loss in advanced CKD should also evaluate the proportion of patients achieving successful kidney transplantation waitlisting, or at least report the number of patients who are no longer excluded because of obesity.

Patients with obesity, T2DM and CKD are an important subgroup to target with intensification of therapy given the progressive nature of DKD. In a longitudinal study of patients with type 2 DKD, annual decline in CKD‐EPI eGFR decreased from −5.6 to −3.1 mL/min/BSA/year after intensification of therapy through an integrated nephrology and diabetology clinic.^13^ Thus, significant residual risk of DKD progression exists despite aggressive RAAS blockade and control of metabolic parameters. Most patients with obesity, T2DM and CKD in our cohort were treated with diabetes therapy which promotes weight gain: insulin and sulphonylureas.^39^ Over 25% of patients were treated with a DPP4‐inhibitor, reflecting the common practice to institute treatment with linagliptin, despite modest glycaemic efficacy, in people with T2DM and CKD as it is predominantly faecally eliminated.^40^ Only two patients were treated with a GLP1RA and none were treated with an SGLT2i. The current study was conducted prior to the publication of the CREDENCE trial,^12^ which will likely increase the proportion of such patients treated with SGLT2is in our practice.

Nevertheless, median eGFR in our study cohort was 29 mL/min/BSA; SGLT2is are not yet licensed in this setting due to concerns that they may lack glycaemic efficacy in those with eGFR <45 mL/min/BSA.^41^ In addition, SGLT2is and loop diuretics have synergistic natriuretic effects, raising the possibility that concerns about the risk of volume depletion in patients already treated with loop diuretics may have limited usage of SGLT2is in our study cohort.^42^, ^43^ That said, patients with CKD are frequently diuretic‐resistant and hence sequential, combined SGLT2i and loop diuretic therapy may be of therapeutic benefit.^44^ Results of the RECEDE‐CHF clinical trial will guide the usage of SGLT2is in patients on loop diuretics.^45^ Although liraglutide does not require dose adjustment with impaired glomerular filtration, there is limited clinical experience with its use in people with advanced CKD, which is reflected in our study findings. Liraglutide does appear to be safe and efficacious from a glycaemic and weight management perspective in people with advanced CKD, although slower titration may be advisable to minimize the incidence of gastrointestinal effects.^46^ Most people with obesity, T2DM and CKD in our centre were not treated with weight‐neutral or weight‐lowering diabetes therapy. Multi‐disciplinary intervention by nephrologists, diabetologists and bariatric physicians in people with obesity and CKD may increase the usage of GLP1RAs and metabolic surgery for glycaemia and weight management indications. The impact of such a strategy on CKD progression and cardiovascular mortality should be investigated.

Our data should be interpreted taking account of certain limitations. The cross‐sectional nature of this study hampered attempts to infer causality. The predominance of white Caucasians in our cohort limits the applicability of our findings to adults from other racial and ethnic backgrounds. The extent to which our findings are generalizable requires confirmation in international cohorts where greater ethnic diversity amongst patients with CKD is observed, particularly given the higher risk of CKD and ESKD amongst African Americans.^47^ Misclassification of individuals as being affected by obesity using BMI as the sole measure of body composition is well described.^48^ This phenomenon may be more common in people with CKD as the accompanying changes in extracellular fluid volume alter body weight independently of adiposity.^49^ We excluded certain individuals from our cohort, including women during pregnancy and the post‐partum period, to minimize the impact of such misclassification on our study findings. Differences in the burden of cardiovascular comorbidities and CKD severity across obesity stages may have been more readily apparent if patients were stratified on a measure of abdominal adiposity. Waist circumference and/or waist‐to‐height ratio should be measured in future studies to assess the impact of obesity in nephrology practice. Given the aforementioned changes in fluid status in CKD, accurate delineation of lean and fat mass using DEXA scanning or bioimpedance should also be considered.^50^

## 5 CONCLUSIONS

In conclusion, obesity was common amongst individuals attending nephrology clinics. Class 1 obesity was more common in males; normal weight and obesity classes 2 and 3 were more frequently observed in females. The severity of obesity did not significantly influence the prevalence of cardiovascular complications or CKD stage amongst people with obesity and CKD. Increasing BMI associated with increasing proteinuria amongst people with obesity and CKD, a relationship which was stronger in males and those with CKD stages 4 and 5. Increasing BMI also associated with greater antihypertensive usage in patients with obesity and CKD. The majority of people with obesity, T2DM and CKD were treated with medications that promote weight gain, including sulphonylureas and insulin; GLP1RAs and SGLT2is were infrequently prescribed in this setting. Prospective studies of medical and surgical intentional weight loss strategies in people with obesity and CKD evaluating renal, cardiovascular, and mortality outcomes are warranted.

## CONFLICT OF INTEREST

ClR discloses personal fees outside of the submitted work from Novo Nordisk, GI Dynamics, Eli Lilly, Johnson and Johnson, Sanofi, Aventis, Astra Zeneca, Janssen, Bristol‐Myers Squibb, and Boehringer‐Ingelheim. The authors declare that they have no competing interests.

## DATA AVAILABILITY

The anonymised dataset used during the current study is available from the corresponding author on reasonable request. Statistical code written in the R programming language for the current study is also available from the corresponding author on reasonable request.

## AUTHOR CONTRIBUTIONS

William P. Martin, Neil G. Docherty, Carel W. le Roux and John Holian designed the study. William P. Martin, Aisling O'Riordan, Alan J. Watson and John Holian provided clinical care to the study cohort. William P. Martin, John Coleman and Ludmilla Dellatorre‐Teixeira collected BMI data for the study cohort. William P. Martin, Jessica Bauer and John Coleman collected clinical and laboratory information for the study cohort, with assistance from Janice L.V. Reeve. Janice L.V. Reeve and Patrick J. Twomey quality assured the laboratory analysis. William P. Martin performed the statistical analyses. William P. Martin, Neil G. Docherty, Aisling O'Riordan, Alan J. Watson, Carel W. le Roux and John Holian interpreted the data. William P. Martin, Neil G. Docherty, Carel W. le Roux and John Holian drafted the manuscript with critical input from all authors. All authors reviewed and approved the final version of the manuscript.
